# Caso 6/2020 – Jovem de 16 Anos, com Estenose Pulmonar Acentuada a Nível Valvar, após Correção de Tronco Arterial Comum pela Técnica de *Barbero-Marcial* no Primeiro Mês de Vida

**DOI:** 10.36660/abc.20190490

**Published:** 2020-09-11

**Authors:** Edmar Atik, Miguel Barbero-Marcial

**Affiliations:** Clínica privada do Dr Edmar Atik Brasil Clínica privada do Dr Edmar Atik

**Keywords:** Cardiopatias Congênitas, Insuficiência Cardíaca, Tronco Arterial/cirurgia, Técnica de Barbero-Marcial, Diagnóstico por Imagem

## Dados Clínicos

O recém-nascido, em insuficiência cardíaca com tronco arterial comum tipo I, foi corrigido com 15 dias de vida com 2.800 g de peso corporal pela técnica de *Barbero-Marcial* . Na ocasião fez-se a aproximação direta da via de saída do ventrículo direito (VD) com o tronco pulmonar e colocação de uma monocúspide em posição pulmonar.

A evolução foi adequada com o controle da insuficiência cardíaca e se manteve sem sintomas e com desenvolvimento físico normal. O exame clínico descartava lesões residuais como a insuficiência valvar pulmonar. No decorrer do tempo, assintomático, observou-se presença de sopro sistólico na área pulmonar, progressivo em intensidade ao lado de gradiente de pressão crescente na região da monocúspide pulmonar. Com 2 anos ele era de 25 mmHg, com 5 anos de 34 mmHg, com 7 anos de 40, com 13 anos de 90 e com 16 anos de 149 mmHg. Não usa medicamentos específicos.

**Exame físico:** bom estado geral, eupneico, acianótico, pulsos normais. Peso: 60 Kg, Alt.: 165 cm, PA: 110/70 mmHg, FC: 73 bpm. A aorta não era palpada na fúrcula.

No precórdio, *ictus cordis* não palpado e sem impulsões sistólicas na BEE. As bulhas cardíacas eram hiperfonéticas e auscultava-se sopro sistólico, +/++/4 de intensidade, rude, na área pulmonar e ao longo da BEE. Fígado não palpado e pulmões limpos.

## Exames Complementares

**Eletrocardiograma:** Mostrava ritmo sinusal e sinais de bloqueio completo do ramo direito. AQRS= +160^o^, AP e AT= 50^o^ C. A duração do QRS era de 0,13”. Não havia potenciais de ventrículo esquerdo, com morfologia rR’ em V1 e RS em V6.

**Figura 1:**
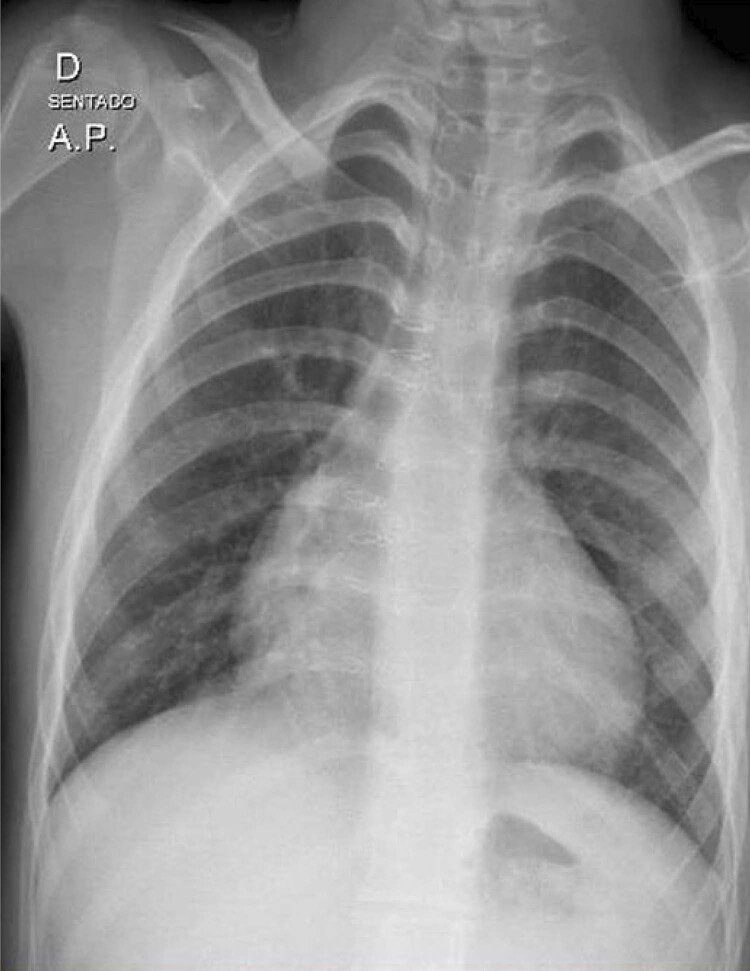
– Radiografia de tórax salienta o aumento moderado da área cardíaca à custa das cavidades direitas com trama vascular pulmonar normal.

**Radiografia de tórax:** Mostra área cardíaca moderadamente aumentada à custa dos arcos atrial e ventricular e trama vascular pulmonar normal. A cardiomegalia foi progressiva desde a correção cirúrgica, com índice cardiotorácico atual de 0,60 ( Figura 1 ).

**Ecocardiograma:** Mostra o remendo interventricular bem posicionado e sem *shunt* residual. As cavidades direitas se mostram dilatadas em grau moderado e com disfunção ventricular. O VD está também hipertrofiado. Gradiente máximo entre VD e tronco pulmonar de 149 mmHg e médio de 86 mmHg. As dimensões eram: Ao=32, AE= 28, VD= 34, VE= 41, septo= parede posterior= 7, função VE= 66%, APD= 22 e APE= 26 mm. Insuficiência pulmonar discreta.

**Tomografia do coração:** Mostrou os átrios de dimensões normais, VD com hipertrofia médio-apical e VDFVD= 135,2 ml/m^2^ e disfunção de VD= 28%. A via de saída do VD mostrava uma monocúspide calcificada e na planimetria da região a abertura valvar era de 0,95 cm^2^, com diâmetro de 14,3 x 6,2 cm. O septo interventricular estava íntegro e a aorta tinha calibre normal. Medidas de interesse: 1) Raiz de aorta: 35,4 x 35,0 mm (Z-score 3,3). 2) Aorta ascendente: 27,6 x 25,2 mm. 3) Arco aórtico proximal: 22,1 x 20,4 mm - médio: 23,6 x 17,6 mm - distal: 21,0 x 17,6 mm. 4) Aorta descendente: - proximal: 13,9 x 13,5 mm - transição tóraco-abdominal: 11,4 x 9,6 mm. 5) Tronco pulmonar: 17,1 x 13,6 mm (Z-score -2,27). 6) Artéria pulmonar direita:16,3 x 13,0 mm (Z-score 0,35). 7) Artéria pulmonar esquerda: 11,7 x 10,1 mm (Z-score -0,96). 9) Ventrículo esquerdo: - Fração de ejeção: 49% - Volume diastólico final indexado: 82,4ml/m^2^.

**Diagnóstico clínico:** Tronco arterial comum tipo I operado precocemente sob a técnica de *Barbero-Marcial* com estenose pulmonar acentuada e progressiva observada na juventude, em paciente assintomático.

**Raciocínio clínico:** Os elementos clínicos evolutivos eram compatíveis com diagnóstico de estenose pulmonar que se instalou progressivamente desde a correção do defeito de base, o tronco arterial comum. A ausência de sintomas era esperada em presença da instalação insidiosa da obstrução através do tempo. A progressão maior da estenose ocorreu nos últimos três anos, provavelmente pela maior calcificação da monocúspide nesse período.

**Diagnóstico diferencial:** Lesão da valva pulmonar após correção cirúrgica pode ocorrer em toda situação na qual a valva pulmonar seja anteriormente reparada. Seu diagnóstico é simples através da presença de sopro sistólico na área pulmonar, acrescentado da hipertrofia miocárdica de VD nos exames de imagem.

**Conduta:** Em face da progressão do defeito residual a nível da valva pulmonar, já com caracteres adquiridos como hipertrofia miocárdica e disfunção de VD, a conduta de intervenção na região obstruída foi facilmente assimilada. Dada a anatomia adequada da região da valva pulmonar com diâmetro de 14 mm e sem dilatação da via de saída do VD, imaginou-se pertinente a abordagem da mesma por cateterismo cardíaco intervencionista. A colocação de prótese valvar tipo *Melody* foi a técnica de escolha, com o inconveniente da possibilidade de ocorrência de endocardite infecciosa em uma valva de origem de veia jugular bovina. As artérias coronárias bem afastadas da via de saída do VD favoreceram a suposição traçada.

**Comentários:** A técnica de *Barbero-Marcial* ^1^ para correção do tronco arterial comum, desenvolvida nos idos de 1989, é habitualmente acompanhada de insuficiência valvar pulmonar em face da dilatação da via de saída do VD na anastomose com o tronco pulmonar, tracionado em direção à mesma. Acompanha ainda a colocação de uma monocúspide, que analogamente ao que ocorre após a correção da tetralogia de *Fallot* , favorece também à evolução posterior de regurgitação pulmonar progressiva. Esses pacientes necessitam de correção do defeito residual e quase sempre por intervenção cirúrgica, em face da grande dilatação da região, que impossibilita a colocação de prótese endovenosa.

A preservação da via de saída mais estreita, como observada no caso apresentado, traz à baila a imaginação e discussão de como deveria se suceder em pacientes semelhantes operados, como mais comumente na tetralogia de *Fallot* . Tal fato poderia ocorrer mais vezes, desde que o cirurgião preservasse mais a via de saída do VD em uma área mais estreita, a permitir que evolutivamente a estenose pulmonar predominasse sobre a insuficiência valvar. Essa preferência decorre do fato de que a lesão miocárdica de sobrecarga de volume seja mais deletéria do que a estenose pulmonar, esta provocada mais pela calcificação da monocúspide.

O ideal é que esses pacientes sejam sempre devidamente monitorados, a fim de se preservar a condição preconizada para evolução mais favorável a mais longo prazo.

**Figura 2:**
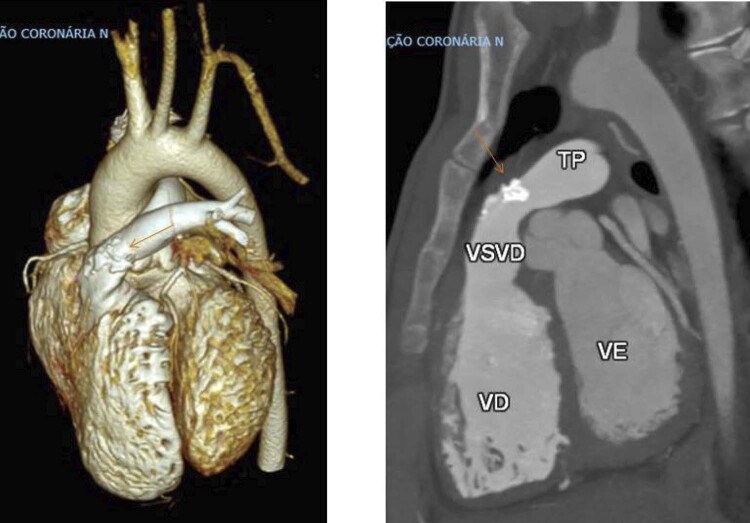
– Angiotomografia do coração em projeções de quatro cavidades e transversal salienta a hipertrofia miocárdica do VD e a via de saída do VD sem dilatação, mas com valva monocúspide nitidamente calcificada (setas). TP: tronco pulmonar; VD: ventrículo direito; VE: ventrículo esquerdo; VSVD: via de saída do ventrículo direito.
