# Supplementary material for: Caso 6/2020 – Jovem de 16 Anos, com Estenose Pulmonar Acentuada a Nível Valvar, após Correção de Tronco Arterial Comum pela Técnica de Barbero-Marcial no Primeiro Mês de Vida
Source: Arq Bras Cardiol. 2020 Sep 11;115(1 Suppl 1):37–9. [Article in Portuguese] doi: 10.36660/abc.20190490 (PMC8386958; doi:10.36660/abc.20190490)
Supplement: Translation

# Translation: Case 6/2020 – 16-Year-Old Adolescent with Severe Pulmonary Stenosis At Valvar Level, After Correction of *Truncus* Arteriosus using the Barbero-Marcial Technique in the First Month of Life

**Authors:** Edmar Atik, Miguel Barbero-Marcial

**Affiliations:** Dr Edmar Atik private clinic Dr Edmar Atik private clinic

**Keywords:** Heart Defects,Congenital, Heart Failure, Truncus Arteriosus/surgery, Barbero-Marcial Procedure, Diagnostic, Imaging

## Clinical Data

The newborn, in heart failure with *truncus arteriosus* type I, underwent repair at 15 days of age, weighing 2800 g. of body weight, by the Barbero-Marcial technique. At that time, the right ventricular outflow tract was approached directly with the pulmonary trunk and a monocusp valve was placed in the pulmonary position.

The evolution was adequate, with heart failure control, and he remained asymptomatic and showed normal physical development. The clinical examination ruled out residual lesions, such as pulmonary valve insufficiency. Over time, while asymptomatic, a systolic murmur was identified in the pulmonary area, of progressive intensity, together with an increasing pressure gradient in the region of the pulmonary monocusp. At 2 years of age, it was 25 mmHg; at 5 years, 34 mm Hg; at 7 years, it was 40; at 13 years it was 90 and at 16 years it was 149 mmHg. The patient did not use any specific medications.

**Physical Examination:** good overall status, eupneic, acyanotic, normal pulses. Weight: 60 Kgs, Height: 165 cm, BP: 110/70 mm Hg, HR: 73 bpm. The aorta was nonpalpable at the suprasternal notch. In the precordium, the apical impulse was nonpalpable and there were no systolic impulses in the left sternal border (LSB). The heart sounds were hyperphonetic and a +/++/4 rough systolic murmur was auscultated in the pulmonary area and along the LSB. Nonpalpable liver and clear lungs.

## Complementary Examinations

**Electrocardiogram** showed sinus rhythm and signs of complete right bundle-branch block. AQRS = +160^o^, AP and AT = 50^o^C. The QRS duration was 0.13”. There were no left ventricular potentials, with rR’ morphology in V1 and RS in V6.

**Chest x-ray** showed moderately increased cardiac area on account of the atrial and ventricular arches and normal pulmonary vascular network. Cardiomegaly was progressive since the surgical correction, with a current cardiothoracic index of 0.60 ( figure 1 ).

**Figure 1:** – Chest x-ray highlights the moderate increase in the cardiac area on the account of the right cavities, with normal pulmonary vascular network.

**Figure 2:** – Angiotomography of the heart in four-cavity and cross-sectional views, highlighting myocardial hypertrophy of the right ventricle and the right ventricular outflow tract without dilation but with a clearly calcified monocusp valve (arrows). Abbreviations: PT: pulmonary trunk, RV: right ventricle, LV: left ventricle, RVOT: right ventricular outflow tract.

**Echocardiogram** showed a well-positioned interventricular patch and no residual shunt. The right cavities were moderately dilated and showed ventricular dysfunction. The RV also showed hypertrophy. The maximum gradient between the RV and the pulmonary trunk was 149 mmHg, with an average of 86 mmHg. The dimensions were: Ao = 32, LA = 28, RV = 34, LV = 41, septum = posterior wall = 7, LV function = 66%, RPA = 22 and LPA = 26 mm. Mild pulmonary insufficiency.

**Cardiac tomography** showed normal-sized atria, right ventricle with medio-apical hypertrophy and RVEDV = 135.2 mL/ m^2^and RV dysfunction = 28%. The RV outflow tract showed a calcified monocusp and the planimetry of the region showed the valve opening was 0.95 cm^2^, with a diameter of 14.3 x 6.2 cm. The interventricular septum was intact, and the aorta had a normal caliber. Measures of interest: 1) Aortic root: 35.4 x 35.0mm (Z-score 3.3). 2) Ascending aorta: 27.6 x 25.2mm. 3) Proximal aortic arch: 22.1 x 20.4mm - mean: 23.6 x 17.6mm - distal: 21.0 x 17.6mm 4) Descending aorta: - proximal: 13.9 x 13.5mm - thoraco-abdominal transition: 11.4 x 9.6mm. 5) Pulmonary trunk: 17.1 x 13.6 mm (Z-score -2.27). 6) Right pulmonary artery: 16.3 x 13.0 mm (Z-score 0.35). 7) Left pulmonary artery: 11.7 x 10.1 mm (Z-score -0.96). 9) Left ventricle: - Ejection fraction: 49% - Indexed end-diastolic volume: 82.4 mL / m^2^.

**Clinical Diagnosis:***Truncus arteriosus* Type I submitted to an early operation using the Barbero-Marcial technique, with severe and progressive pulmonary stenosis observed in adolescence, in an asymptomatic patient.

**Clinical reasoning:** The evolution clinical elements were compatible with the diagnosis of progressive pulmonary stenosis since the correction of the basal defect, the *Truncus arteriosus* Type I. The absence of symptoms was expected in the presence of the insidious occurrence of the obstruction over time. The greatest progression of stenosis had occurred in the last three years, probably due to the greater calcification of the monocusp during this period.

**Differential diagnosis:** Pulmonary valve injury after surgical correction can occur in any situation in which the pulmonary valve is previously repaired. Its diagnosis is simple, attained through the presence of a systolic murmur in the pulmonary area, plus right ventricular myocardial hypertrophy in imaging exams.

**Conduct:** Considering the progression of the residual defect at the pulmonary valve level, with acquired characteristics such as myocardial hypertrophy and right ventricular dysfunction, the intervention approach in the obstructed region was easily assimilated. Given the adequate anatomy of the pulmonary valve region, with a diameter of 14 mm and without RV outflow tract dilation, it was considered pertinent to approach it using interventional cardiac catheterization. The use of a Melody prosthetic valve was the technique of choice, with the inconvenience of the possibility of occurrence of infectious endocarditis in a bovine jugular vein valve. The fact that the coronary arteries were well away from the right ventricular outflow tract favored the established assumption.

**Comments:** The use of the Barbero-Marcial^1^technique for correction of the *truncus arteriosus* Type I, developed in 1989, is usually accompanied by pulmonary valve insufficiency due to the RV outflow tract dilation at the anastomosis with the pulmonary trunk, which is pulled towards it. It also accompanies the placement of a monocusp, which, analogously to what occurs after the correction of the Tetralogy of Fallot, also favors the subsequent evolution of progressive pulmonary regurgitation. These patients require correction of the residual defect and almost always by surgical intervention, due to the large dilation in the region, which makes it impossible to place an intravenous prosthesis.

The preservation of the narrower outflow tract, as observed in the case presented herein, brings to mind the occurrence and discussion of how it should happen in similar operated patients, as most commonly in the Tetralogy of Fallot. This fact could occur more frequently, as long as the surgeon better preserved the RV outflow tract in a narrower area, allowing the pulmonary stenosis evolution to predominate over valve regurgitation. This preference stems from the fact that the volume overload myocardial lesion is more harmful than pulmonary stenosis, which is caused more often by monocusp calcification.

Ideally, these patients should always be adequately monitored, aiming to preserve the recommended condition for a more favorable evolution in the longer term.
